# Effects of dietary supplementation of leaves and whole plant of *Andrographis paniculata* on rumen fermentation, fatty acid composition and microbiota in goats

**DOI:** 10.1186/s12917-017-1223-0

**Published:** 2017-11-24

**Authors:** Aisha L. Yusuf, Kazeem D. Adeyemi, Anjas A. Samsudin, Yong M. Goh, Abdul Razak Alimon, Awis Q. Sazili

**Affiliations:** 1 Department of Animal Science, Faculty of Agriculture, Universiti Putra Malaysia, 43400 UPM Serdang, Selangor Darul Ehsan Malaysia; 2 Laboratory of Sustainable Animal Production and Biodiversity, Institute of Tropical Agriculture and Food Security, Universiti Putra Malaysia, 43400 UPM Serdang, Selangor Darul Ehsan Malaysia; 3 Halal Products Research Institute, Putra Infoport, Universiti Putra Malaysia, 43400 UPM Serdang, Selangor Darul Ehsan Malaysia; 4 Department of Veterinary Preclinical Sciences, Faculty of Veterinary Medicine, Universiti Putra Malaysia, 43400 UPM Serdang, Selangor Darul Ehsan Malaysia; 5 Department of Animal Science, Usmanu Danfodiyo University, P.M.B, Sokoto, 2346 Nigeria; 6 Department of Animal Production, University of Ilorin, Ilorin, Nigeria

**Keywords:** Nutrient digestibility, *Ruminococcus albus*, *Ruminococcus flavefaciens*, *Fibrobacter succinogenes*

## Abstract

**Background:**

The nature and amount of dietary medicinal plants are known to influence rumen fermentation and nutrient digestibility in ruminants. Nonetheless, changes in nutrient digestibility and rumen metabolism in response to dietary *Andrographis paniculata* (AP) in goats are unknown. This study examined the effects of dietary supplementation of leaves and whole plant of AP on nutrient digestibility, rumen fermentation, fatty acids and rumen microbial population in goats. Twenty-four Boer crossbred bucks (4 months old; average body weight of 20.18 ± 0.19 kg) were randomly assigned to three dietary groups of eight goats each. The dietary treatments included a control diet (Basal diet without additive), basal diet +1.5% (*w*/w) *Andrographis paniculata* leaf powder (APL) and basal diet +1.5% (w/w) *Andrographis paniculata* whole plant powder (APW). The trial lasted 100 d following 14 d of adjustment.

**Results:**

The **r**umen pH and concentration of propionate were greater (*P* < 0.05) in goats fed the APL and APW diets than those fed the control diet. The concentrations of ammonia nitrogen and acetate were greater (*P* < 0.05) in the control goats than the APL and APW goats. The digestibilities of crude protein, dry matter, acid detergent fibre and neutral detergent fibre were greater (*P* < 0.05) in the APL and APW goats compared to the control goats. Dietary APL and APW decreased (*P* < 0.05) the ruminal concentration of C18:0 and increased (*P* < 0.05) the ruminal concentration of C18:2n-6 and C18:3n-3. The APL goats had greater (*P* < 0.05) ruminal concentration of C18:1 *trans*-11 and CLA *cis*-9 *trans*-11 than the APW and control goats. Dietary treatments had no significant effect on the population of protozoa and methanogens in the rumen of goats. The ruminal populations of *Ruminococcus albus*, *Ruminococcus flavefaciens* and *Fibrobacter succinogenes* were greater (*P* < 0.05) in the APL and APW goats than the control goats.

**Conclusion:**

Dietary supplementation of leaves and whole plant of *Andrographis paniculata* can be used to manipulate rumen metabolism for improved nutrient digestibility in goats.

## Background

The manipulation of the rumen ecosystem is an important strategy for ensuring efficient feed utilization in ruminants [1, 2]. Improved feed efficiency could help to achieve the desired production targets with minimal negative impact on the environment [2, 3]. Plant secondary metabolites (PSM) in medicinal plants are capable of manipulating rumen metabolism and this could have environmental and production merits or demerits [2–6]. The major factors influencing the effects of PSMs in ruminant nutrition are the chemical nature of PSMs, their concentration in diet and the diversity and abundance of rumen microbiota [2–6].

A plethora of studies has examined the impact of supplementing myriad PSMs on rumen metabolism and nutrient digestibility in ruminants [2, 3, 5, 6]. However, there are wide variations in outcome between different studies. Therefore, there is need for additional studies in different production systems to permit tailored decisions and informed choices in the utilization of PSMs in ruminant nutrition.

*Andrographis paniculata* (AP), called ‘king of bitters’ is a medicinal plant that is native to Asia [7]. The PSMs in different parts of AP [7–9] and the effects of AP on in vitro rumen metabolism [9] and growth performance in goats [10] have been documented. However, information regarding the effects of AP on in vivo rumen metabolism and nutrient digestibility in ruminants is very scarce. In addition, the response of rumen microbiota to dietary AP is unknown. Thus, the objective of this study was to determine the effects of dietary AP on nutrient digestibility, rumen fermentation and the population of rumen microbiota in goats.

## Methods

### Animals and diets

Twenty-four Boer bucks (4 months old, with average initial body weight of 20.18 ± 0.19 kg) were used for this trial. Each goat was housed in individual pen (1.20 m × 0.80 m × 0.70 m) furnished with drinking and feeding facilities. The goats were treated against endo and ectoparasites prior to the commencement of the trial. The goats were randomly assigned to three dietary groups of eight goats each. The experimental diets were formulated to meet the nutritional requirements of growing goats in line with the NRC [11] recommendations. The dietary treatments included a control diet (basal diet without additive), a basal diet +1.5% (*w*/w) *Andrographis paniculata* leaf powder (APL) and a basal diet +1.5% (w/w) *Andrographis paniculata* whole plant powder (APW). The additives were incorporated into the concentrate portion of the diet. The experimental diets were offered as complete ration mix (forage and concentrate) in two equal meals at 0830 and 1430 h. All goats had ad libitum access to water. The experiment lasted 100 d following two weeks of adaptation.

### Feed analysis

Feed samples (400 g) were collected weekly and stored at −20 °C until analysis. Feed samples were dried at 60 °C for 48 h to determine the DM content, ground to pass through a 1 mm screen and analysed for ash, ether extract and crude protein according to the methods of AOAC [12]. The neutral detergent fibre (NDF) and acid detergent fibre (ADF) were analysed by the protocol of Van Soest et al. [13]. The ingredients and proximate composition of the experimental diets are presented in Table 1 while the fatty acid composition of the dietary treatments is presented in Table 2.

**Table 1:** Ingredients and chemical composition of dietary treatments

	Dietary treatments		
Ingredient (%)	Control	APL	APW
Oil palm frond	40.00	40.00	40.00
Rice husk	10.00	10.00	10.00
Napier grass	10.00	10.00	10.00
Concentrate	40.00	40.00	40.00
APL	0.00	1.50	0.00
APW	0.00	0.00	1.50
Chemical composition (% DM)			
Dry matter	89.86	89.88	89.89
Crude protein	16.79	16.79	16.79
Ether extract	5.89	5.80	5.80
Ash	5.04	5.15	5.16
Acid detergent fibre	30.10	30.94	30.98
Neutral detergent fibre	48.58	48.64	48.56
Calcium	1.41	1.41	1.41
Phosphorus	1.03	1.03	1.03
Metabolizable energy (MJ/kg DM^a^)	10.92	10.90	10.90

*Control* basal diet, *APL* basal diet +1.5% (w/w) *Andrographis paniculata* leaf powder, *APW* basal diet +1.5% (*w*/w) whole plant of *Andrographis paniculata* powder

^a^calculated

**Table 2:** Fatty acid composition (% of total fatty acid) of dietary treatments

	Dietary treatments		
Fatty acid (%)	control	APL	APW
C10:0	0.41	1.11	0.99
C12:0	14.08	5.12	15.20
C14:0	6.90	5.98	5.63
C15:0	0.11	0.06	0.07
C16:0	18.21	17.50	17.42
C16:1	0.23	0.22	0.31
C17:0	0.27	0.19	0.19
C18:0	4.72	4.19	4.43
C18:1 n-9	24.06	24.09	24.05
C18:2 n-6	25.66	26.20	26.25
C18:3 n-3	5.55	5.65	5.49
∑SFA	44.70	44.15	43.93
∑USFA	55.50	55.94	56.07
n6:n3	4.62	4.63	4.78

*Control* basal diet, *APL* basal diet +1.5% (w/w) *Andrographis paniculata* leaf powder, *APW* basal diet +1.5% (w/w) whole plant of *Andrographis paniculata* powder. ΣSFA = (C10:0 + C12:0 + C14:0 + C15:0 + C16:0 + C17:0 + C18:0), ΣUFA = (C16:1+ C18:1 + C18:2n-6 + C18:3n-3), n-6:n-3 = (C18:2n-6÷C18:3n-3)

### Apparent nutrient digestibility trial

The apparent nutrient digestibility trial was conducted between d 70 and 90 of the trial (10 d for adaptation and 10 d for faecal collection). Each animal in each treatment was housed in a metabolic crate measuring 1.20 m × 0.5 m × 0.90 m each, and furnished with drinking and feeding facilities. The daily feed allowance was offered in two equal meals at 0830 and 1430 h. All goats had ad libitum access to water. Daily dry matter intake (DMI) was estimated as the arithmetic difference between the offered and refused dry matter. Daily feces from individual animal was collected and weighed in the morning before feeding. After thorough mixing, approximately 50 g/Kg of the daily feces from each animal were sampled and stored at −20 °C for subsequent analyses.

### Collection of rumen fluid

About 50 mL of rumen liquor was collected from each goat after 2 h of feeding by aspiration using stomach tube. The rumen fluid pH was immediately measured using the Mettler-Toledo pH meter. The rumen fluid was strained through four layers of cheesecloth and 5 mL of 1 M H_2_SO_4_ was added. The rumen fluid was snap frozen in liquid nitrogen and stored at -80^o^ C until further analyses.

### Determination of volatile fatty acids (VFA) in rumen liquor

The rumen liquor was fixed with 25% (*v*/v) metaphosphoric acid and centrifuged at 4000 g. The supernatant (0.5 mL) was collected and added to 0.5 mL of 20 mM valeric acid. The VFA content of the rumen fluid was determined using gas chromatography as described by Yusuf et al. [9]. The sample peaks were identified by comparing with authentic VFA standards.

### Determination of ammonium nitrogen in rumen liquor

Ammonium nitrogen was determined as described by Parsons et al. [14]. A Standard solution was prepared using ammonium chloride (NH_4_Cl) by dissolving 1.908 g of NH_4_Cl in 500 mL distilled water, which gave 1000 mg /L ammonia-N. Thereafter, 0.2, 0.5, 1.0 and 2.0 ppm solutions were prepared by dissolving 0.02, 0.05, 0.10 and 0.20 mL of the stock solution into 100 mL of distilled water, respectively. Approximately 5 mL standard was added into an Erlenmeyer flask containing 0.2 mL phenol solution and swirled. In sequence, 0.2 mL nitroprusside and 0.5 mL of oxidizing solution were added, thoroughly mixed and allowed to stand for 24 h. The absorbance was determined at 640 nm using SC spectrophotometer (Labomed Inc., Culver City, CA, USA). Regression equation was determined from the blank and standard samples and ammonia-N was estimated in the samples.

### Determination of fatty acid (FA) composition

The total FA in feed and rumen liquor was extracted in chloroform: methanol mixture (2:1, *v*/v) following the method Folch et al. [15]. The extracted fat was transmethylated into their fatty acid methyl esters (FAME) using 0.66 N KOH in methanol and 14% methanolic boron trifluoride (BF_3_) according to the protocol of AOAC [12]. The separation of the FAME was done with a gas chromatograph (Model 6890 Agilent Technologies, USA). The gas chromatograph settings and quantification of FA was done as described by Adeyemi et al. [16].

### Extraction of DNA from rumen microbes

Total bacteria DNA in the rumen was extracted using the QIAamp® mini stool kit (QIAGEN, Hilden, GmbH) following the manufacturer’s protocol with slight modification as described by Khaing et al. [17].

### Quantitative real-time PCR

A standard curve method in real-time PCR was used to quantify rumen bacteria, protozoa and methanogenic populations. The standard curves were constructed using the number of copies of the 16S rRNA gene plotted against quantification cycle (Cq) that was obtained from ten-fold serial dilutions of PCR products from a pure culture of each group of rumen microorganism. The DNA was extracted from the pure culture of each targeted rumen microorganisms in order to prepare the standard curves. The bacterial DNA was amplified using conventional PCR. The PCR products of the targeted rumen microorganisms were run in 1% agarose gel and specific bands were purified using the mEGAquick-spinTM purification kit. The concentration and purity of 16S rRNA gene in each sample was determined using a NanoDrop ND-1000 UV-Vis Spectrophotometer (NanoDrop Technologies, Wilmington, DE, USA) at 260/280 nm absorbance. The number of copies of the 16S–rRNA gene per mL of elution buffer was quantified using the formula obtained online in URI Genomics and Sequencing Centre web-based calculator (www.uri.edu/research/gsc/resources/cndna.html).

$$ \mathrm{Number}\  \mathrm{of}\  \mathrm{copies}=\frac{\mathrm{Amount}\  \mathrm{of}\ \mathrm{DNA}\ \left(\upmu \mathrm{g}/\mathrm{mL}\right)\times 6.022\times {10}^{23}}{\mathrm{Length}\ \left(\mathrm{bp}\right)\times {10}^9\times 650} $$

Since the amplification efficiency among templates and primers may be variable, the amplification Efficiency (E) of each primer-template combination was estimated based on the slope value of the linear regression of each standard curve determined by the equation below:

$$ \mathrm{E}\ \left(\%\right)=\left[{10}^{\left(\hbox{-} 1/\mathrm{slope}\right)}\hbox{-} 1\right]\times 100 $$

Where E is 100% if a ten-fold dilution of DNA template results in a Cq difference of 3.32.

The sequences and primers used to quantify the number of different microbes are shown in Table 3. The Real-time PCR was conducted with the Bio-Rad CFX96 Touch (Bio-Rad Laboratories, Hercules, CA, USA) using optical grade plates. The PCR reaction was performed on a total volume of 25 μL using the iQTMSYBR Green Supermix (Bio-Rad Laboratories, Hercules, CA, USA). Each reaction included 12.5 μL SYBR Green Supermix, 1 μL of forward primer (10 μM), 1 μL of reverse primer (10 μM), 2 μL of DNA samples and 8.5 μL RNAse free water. The conditions applied to each well included an initial incubation at 94 °C for 5 min; 40 cycles of denaturation for 20 s at 94 °C annealing for 30 s and for 20 s at 72 °C. A melting curve analysis was conducted after the last cycle of each amplification to confirm the specificity of amplification.

**Table 3:** Microorganisms, sequences and references for the primers used

Microorganism	Sequence 5′ – 3′	Product size (bp)	Annealing temperature (°C)	Reference
Total bacteria F	CGGCAACGAGCGCAACCC	145	55	[18]
Total bacteria R	CCATTGTAGCACGTGTGTAGCC			
Total protozoa F	CTTGCCCTCYAATCGTWCT	223	55	[19]
Total protozoa R	GCTTTCGWTGGTAGTGTATT			
Methanogens (mcrA)-F	TTCGGTGGATCDCARAGRGC	140	55	[20]
Methanogens (mcrA)-R	GBARGTCGWAWCCGTAGAATCC			
*Fibrobacter succinogenes*	GTTCGGAATTACTGGGCGTAAA	122	55	[21]
*Fibrobacter succinogenes*	CGCCTGCCCCTGAACTATC			
*Ruminococcus albus*	CCCTAA AAGCAGTCTTAGTTCG	170	55	[18]
*Ruminococcus albus*	CCTCCTTGCGGTTAGAACA			
*Ruminococcus flavefaciens* F (Rf154f)	TCTGGAAACGGATGGTA	259	55	[18]
*Ruminococcus flavefaciens* (Rf425r)	CCTTTAAGACAGGAGTTTACAA			

*F* Forward, *R* Reverse

### Statistical analysis

The data were analyzed in a completely randomized design using the MIXED procedure of SAS [22] with sampling time as a repeated measure. Dietary treatments, sampling time and the interaction between diet and sampling time were fitted as fixed effects while goats within treatments were fitted as random effects. The real-time PCR data did not meet the ANOVA requirement of normality. Thus, the data was subjected to logarithm transformation prior to subjecting it to the MIXED procedure of SAS [37]. Means were separated using the “PDIFF” option of the “LSMEANS” statement of the MIXED procedure. Dunnett’s test was used to adjust the means. The level of significance difference was set at *P* < 0.05. The statistical model used for the analyses was:

$$ {y}_{ijk}=\mu +{\tau}_i+{\mathrm{d}}_{\mathrm{ij}}+{\mathrm{S}}_{\mathrm{k}}+{\left(\tau \mathrm{S}\right)}_{\mathrm{ik}}+{\varepsilon}_{ijk} $$

Where *y*_*ijk*_ = observation for a parameter.

*μ* = the overall mean.

*τ*_*i*_ = effect of dietary treatment *i.*

d_ij_ = random effect of goats within treatment.

S_k_ = effect of sampling time *k.*

(*τ*S)_ik_ = interaction between dietary treatment and sampling time.

*ε*_*ijk*_ = random error term.

## Results

### Apparent nutrient digestibility

Dry matter intake and digestibility, and apparent nutrient digestibility coefficients in goats fed different parts of AP are shown in Table 4. The digestibilities of DM, CP, NDF and ADF were greater (*P* < 0.05) in the APL and APW goats than the control goats. Dietary AP had no significant effect (*P* > 0.05) on DMI and ether extract digestibility in goats.

**Table 4:** Dry matter intake and nutrient digestibility (Least square means ± standard error of mean) in goats fed diets containing different parts of *Andrographis paniculata*

	Dietary treatments			
Parameter	Control	APL	APW	*P* value
DMI (g/d)	796.33 ± 4.21	816.10 ± 5.23	827.13 ± 4.50	0.08
DM digestibility (%)	61.87 ± 0.04	66.35^+^ ± 0.03	66.50^+^ ± 0.01	0.03
Nutrient digestibility (%)				
Crude protein	43.98 ± 1.57	60.35^+^ ± 1.55	67.50^+^ ± 0.07	0.01
Ether extract	62.71 ± 1.10	64.02 ± 0.19	64.85 ± 0.30	0.64
Acid detergent fibre	39.42 ± 0.24	40.69^+^ ± 0.14	40.89^+^ ± 0.10	0.04
Neutral detergent fibre	57.53 ± 0.20	60.32^+^ ± 0.07	60.50^+^ ± 0.03	0.02

*Control* basal diet, *APL* basal diet +1.5% (*w*/w) *Andrographis paniculata* leaf powder, *APW* basal diet +1.5% (w/w) whole plant of *Andrographis paniculata* powder

^+^Differ from the control (*P* < 0.05)

### Rumen fermentation

The rumen fermentation parameters in goats fed different parts of AP are presented in Table 5. The rumen pH was lower (*P* < 0.05) in the control goats than the APL and APW goats. The concentration of ammonia nitrogen and the acetate/propionate ratio in the control goats were greater (*P* < 0.05) than that of the APL and APW goats. The control goats had greater (*P* < 0.05) concentration of acetate and lower (*P* < 0.05) concentration of propionate than the APL and APW goats. Dietary AP did not affect (*P* > 0.05) the concentration of total VFA and butyrate in the rumen of goats.

**Table 5:** Rumen fermentation profiles (Least square means ± standard error of mean) in goats fed diets containing different parts of *Andrographis paniculata*

	Dietary treatment			
Parameter	Control	APL	APW	*P* value
pH	6.10 ± 0.01	6.25^+^ ± 0.01	6.25^+^ ± 0.01	0.02
Ammonia (mg dL^−1^)	22.05 ± 0.01	19.25^+^ ± 0.42	19.10^+^ ± 0.05	0.02
Total VFA (mM)	63.09 ± 2.44	59.72 ± 1.49	57.99 ± 4.14	0.39
Acetate (mM)	48.84 ± 1.89	38.00^+^ ± 1.10	35.02^+^ ± 1.33	0.01
Propionate (mM)	11.46 ± 0.03	18.44^+^ ± 0.08	20.00^+^ ± 0.06	0.03
Butyrate (mM)	1.68 ± 0.01	1.48 ± 0.01	1.33 ± 0.01	0.09
Acetate/Propionate	4.26 ± 0.02	2.06^+^ ± 0.01	1.75^+^ ± 0.01	0.01

*Control* basal diet, *APL* basal diet +1.5% (*w*/w) *Andrographis paniculata* leaf powder, *APW* basal diet +1.5% (w/w) whole plant of *Andrographis paniculata* powder

^+^Differ from the control (*P* < 0.05)

### Fatty acid composition of rumen liquor

The fatty acid composition of rumen liquor in goats fed different parts of *Andrographis paniculata* is presented in Table 6. The percentages of C10:0, C14:0 and C15:1 in the rumen liquor of the control goats did not differ (*P* > 0.05) from that of the APL goats. The APW goats had lower (*P* < 0.05) percentages of C10:0, C14:0 and C15:1 than the control goats. Dietary supplementation of AP had no significant effect on the percentages of C15:0, C16:0, C17:0 and C18:1n-9 in the rumen of goats. The control goats had greater (*P* < 0.05) percentage of C18:0 than the APL and APW goats. The percentages of C18:1*trans*11 and CLA *cis*-9 *trans*-11 in goats fed APL diet were greater (*P* < 0.05) than those fed the control and APW diets. The control and APW goats had similar percentages of C18:1*trans*11 and CLA *cis*-9 *trans*-11. The APL and APW goats had greater (*P* < 0.05) percentages of C18:2n-6 and C18:3n-3 than the control goats. Ruminal total unsaturated fatty acids was greater (*P* < 0.05) while the total saturated fatty acids and n6:n3 ratio were lower (*P* < 0.05) in the APW goats than the control and APL goats.

**Table 6:** Fatty acid composition (Least square means ± standard error of mean) of rumen liquor in goats fed different parts of *Andrographis paniculata*

Fatty acid (% of total FA)	Dietary treatments			
	Control	APL	APW	*P* value
C10:0	0.06 ± 0.01	0.06 ± 0.02	0.02^+^ ± 0.01	0.02
C12:0	8.55 ± 0.27	6.77^+^ ± 0.29	4.63^+^ ± 0.34	0.01
C14:0	8.86 ± 0.12	8.72 ± 0.17	6.15^+^ ± 0.16	0.01
C14:1	0.70 ± 0.01	0.78^+^ ± 0.03	0.70 ± 0.02	0.02
C15:0	0.61 ± 0.02	0.56 ± 0.02	0.62 ± 0.06	0.78
C15:1	0.27 ± 0.01	0.21 ± 0.04	0.09^+^ ± 0.02	0.01
C16:0	20.39 ± 0.18	20.41 ± 0.49	18.64 ± 1.09	0.33
C16:1	0.51 ± 0.08	0.76^+^ ± 0.10	0.83^+^ ± 0.08	0.04
C17:0	0.44 ± 0.01	0.41 ± 0.04	0.49 ± 0.13	0.11
C18:0	36.64 ± 0.55	32.73^+^ ± 0.57	27.01^+^ ± 1.34	0.01
C18:1n-9	3.53 ± 0.25	4.68 ± 0.89	6.61 ± 2.08	0.21
C18:1*trans*11	2.75 ± 0.12	3.26^+^ ± 0.37	2.65 ± 0.39	0.02
CLA *cis*-9 *trans*-11	0.45 ± 0.01	0.72^+^ ± 0.01	0.51 ± 0.05	0.01
C18:2n-6	1.39 ± 0.17	3.43^+^ ± 0.32	5.26^+^ 0.56	0.03
C18:3n-3	0.18 ± 0.01	0.51^+^ ± 0.04	0.81^+^ ± 0.43	0.01
∑SFA	78.56 ± 0.49	74.66 + 0.76	67.55^+^ 1.88	0.03
∑UFA	21.44 ± 0.49	25.34 ± 0.76	32.45^+^ ± 1.23	0.04
n6:n3	7.72 ± 0.45	7.31 ± 0.22	6.49^+^ ± 0.48	0.02

*Control* basal diet, *APL* basal diet +1.5% (w/w) *Andrographis paniculata* leaf powder, *APW* basal diet +1.5% (w/w) whole plant of *Andrographis paniculata* powder. ΣSFA = (C10:0 + C12:0 + C14:0 + C15:0 + C16:0 + C17:0 + C18:0), ΣUFA = (C14:1 + C16:1+ C18:1 + C18:2n-6 + C18:3n-3), n-6:n-3 = (C18:2n-6÷C18:3n-3)

^+^Differ from control (*P* < 0.05)

### Rumen microbial populations

The response of rumen microbial populations to dietary supplementation of AP is presented in Table 7. Dietary AP had no significant effect on the populations of protozoa, methanogens and total bacteria in the rumen of goats. The populations of *Ruminococcus albus, Ruminococcus flavefaciens* and *Fibrobacter succinogenes* were greater (*P* < 0.05) in the APW and APL goats than the control goats.

**Table 7:** Rumen microbial profile (Least square means ± standard error of mean) in goats fed diets containing different parts of *Andrographis paniculata*

Parameters	Log_10_ cell mL^−1^			
	Dietary treatments			
	Control	APL	APW	*P* value
Total bacteria	10.57 ± 0.20	10.83 ± 0.22	10.97 ± 0.21	0.45
Total methanogens	5.50 ± 0.66	5.01 ± 0.81	4.36 ± 0.66	0.23
*Ruminococcus albus*	6.81 ± 0.45	8.08^+^ ± 0.53	8.38^+^ ± 0.47	0.03
*Ruminococcus flavefaciens*	5.46 ± 0.21	5.98^+^ ± 0.23	6.55^+^ ± 0.22	0.04
*Fibrobacter succinogenes*	3.88 ± 0.25	4.50^+^ ± 0.28	4.71^+^ ± 0.23	0.04
Total protozoa	5.82 ± 0.52	5.49 ± 0.61	5.31 ± 0.55	0.12

*Control* basal diet, *APL* basal diet +1.5% (w/w) *Andrographis paniculata* leaf powder, *APW* basal diet +1.5% (w/w) whole plant of *Andrographis paniculata* powder

^+^Differ from the control (P < 0.05)

## Discussion

The aim of this study was to determine the effects of dietary supplementation of leaves and whole plant of *Andrographis paniculata* on nutrient digestibility and rumen metabolism in goats. It was evident that dietary supplementation of APW and APL enhanced apparent nutrient digestibility in goats. The increased DM, NDF and ADF digestibilities could be due to the increase in the populations of *Ruminococcus albus*, *Ruminococcus flavefaciens* and *Fibrobacter succinogenes* in the rumen of goats fed AP diets. The increase in CP digestibility in the AP-supplemented goats could be due to the tannin present in AP [9], which protected the dietary protein from ruminal degradation. This observation lends credence to the lower concentration of ammonia nitrogen in the rumen of goats fed the AP diets. Similarly, dietary supplementation of various medicinal herbs enhanced nutrient digestibility in goats [23] and lambs [6]. Contrarily, dietary supplementation of Pakar (*Ficus infectoria*) leaves reduced nutrient digestibility in goats [24]. The similarity in the DMI of the goats suggests that AP did not reduce feed palatability in goats.

The ruminal concentration of ammonia nitrogen is an effective indicator of microbial activity in the rumen [17]. The minimum concentration of ammonia nitrogen needed for microbial protein synthesis is 5 mg dL^−1^ [25] while ammonia nitrogen concentration in the range of 10–20 mg dL^−1^ is needed for optimum fibre degradation in the rumen [26]. The level of ammonia nitrogen observed in the current study exceeded 5 mg dL^−1^ and ranged between 19.10 and 22.05 mg dL^−1^. This observation indicates that the concentrations of ammonia nitrogen observed following the supplementation of the dietary treatments were adequate for optimum microbial growth and rumen fermentation.

Dietary supplementation of AP reduced the concentration of ammonia nitrogen in the rumen of goats. This observation attests to the ability of AP polyphenols to protect dietary protein from microbial degradation. The current finding concurs with that of Singh et al. [24] who found a significant reduction in the concentration of ammonia nitrogen in goats fed Pakar leaves compared with those fed the basal diet. Similarly, dietary *Lotus corniculatus* [27] and sainfoin [28] reduced the concentration of ammonia nitrogen in the rumen of sheep. However, dietary Oak (*Quercus libani* Oliv.) did not affect the concentration of ammonia nitrogen in sheep [29].

The pH is an effective indicator of rumen metabolism [17]. The rumen pH observed in the current study ranged from 6.10 to 6.25, and it is ideal for optimum rumen metabolism [17]. The APL and APW goats had greater rumen pH than the control goats. This finding could be due to the increase in the production of saliva and/or changes in saliva composition induced by the tannins present in the AP. Saliva serves as an important buffering capacity in the rumen [30]. An increase in salivary flow has been associated with the consumption of tanniferous feeds. For instance, Salem et al. [31] reported that the consumption of quebracho tannins increased the quantity of parotid saliva produced by goats and sheep as the supplementation period progressed. Contrary to the current observation, dietary Oak [29] and Pakar leaves [24] had no effect on rumen pH in sheep and goats respectively.

Dietary AP did not affect the total VFA in the rumen of goats. A similar observation was reported following dietary supplementation of Pakar leaves in goats [24]. Contrarily, condensed tannin reduced total VFA, while mangosteen peel [32] and rain pod tree [33] increased total VFA when supplemented. Dietary AP increased the concentration of propionate and reduced the concentration of acetate in the rumen of goats. This observation agrees with the report of earlier studies wherein, the supplementation of mangosteen peel [32] and rain pod tree [33] increased the concentration of propionate and decreased the concentration of acetate.

Regardless of the dietary treatments, the most abundant FA in the rumen liquor of goats was C18:0 followed by C16:0. Similar findings were obtained in the in vitro [9] and in vivo rumen FA profile of goats [34]. Dietary supplementation of AP reduced the percentage of C18:0 in the rumen liquor of goats. Stearic acid is the final product of biohydrogenation of unsaturated fatty acids [35]. The lower proportion of C18:0 in the rumen of goats fed AP diets could be due to the selective antimicrobial activity of AP [7, 8], which hindered the growth and/or metabolism of microbes responsible for the complete biohydrogenation of unsaturated FA. It has been reported that the activities of *Butyrovibrio proteoclasticum,* the bacterium responsible for the biosynthesis of stearic acid from linoleic acid could be weakened by polyphenols, which have the capacity to bind microbial cell membrane, protein and enzymes [36]. The current finding is consistent with those of Khiaosa-Ard et al. [37] and Vasta et al. [38] who observed that tanninferous diets reduced ruminal concentration of C18:0.

Dietary APL and APW enhanced the percentage of C18:2n-6 and C18:3n-3 in the rumen liquor of goats. This observation suggests that AP reduced ruminal lipolysis of polyunsaturated fatty acids (PUFA) in goats. Polyphenols can bind both plant and microbial lipases and inhibit lipolysis and biohydrogenation of PUFA [39]. Jayanegara et al. [40] observed that phenolic compounds in tropical forages inhibited the in vitro biohydrogenation of C18:2n-6 and C18:3n-3 and reduced the production of C18:0. Contrarily, the supplementation of quebracho tannins did not affect the disappearance of C18:2n-6 and C18:3n-3 [5]. The APL goats had greater concentration of biohydrogenation intermediates (C18:1 *trans*-11 and CLA *cis*-9 *trans*-11) than the APW and control goats. This suggests that APL and APW differ in their effects on ruminal lipolysis and biohydrogenation of PUFA, possibly due to the differences in the polyphenol contents [9]. The effects of polyphenol on the concentration of biohydrogenation intermediates in the rumen have yielded inconsistent results in the published literature. The discrepancies were attributed to the chemical nature of polyphenols, intricate interactions between dietary components and the polyphenols and the adaptation of microbes to the polyphenols [4, 5]. In addition, it has been suggested that the impact of polyphenols on biohydrogenation of unsaturated fatty acids depends on the biohydrogenation step that they affect [5]. Thus, it could be inferred that the APW diet exhibited greater inhibition on lipolysis and biohydrogenation of unsaturated fatty acids hence its lower concentration of biohydrogenation intermediates and C18:0 and greater concentration of C18:2n-6 and C18:3n-3 when compared with the APL diet. The increase in the concentration of C18:1 *trans*-11 and CLA *cis*-9 *trans*-11 in the APL goats is consistent with the findings of Vasta et al. [5] who observed that the supplementation of quebracho tannins increased the ruminal concentration of C18:1 *trans*-11 in sheep. Similarly, phenolic compounds in tropical forages enhanced the appearance of CLA *cis*-9 *trans*-11 but not C18:1 *trans*-11 [40].

Dietary AP had no significant effect on the populations of protozoa and methanogens in the rumen of goats. This finding suggests that the level of polyphenols in the AP diets [9] was not sufficient to reduce the populations of protozoa and methanogens. The response of protozoa and methanogens to the supplementation of medicinal herb has yielded inconsistent results in the published literature. Singh et al. [24] reported a reduction in rumen protozoa counts and an increase in methanogen counts in goats fed Pakar leaves. Tan et al. [41] observed that tannins in *Leucaena leucocephala* reduced the populations of protozoa and methanogens in vitro. Baah et al. [42] revealed that quebracho tannins reduced ruminal protozoa in heifers. However, Khiaosa-Ard et al. [37] observed that dietary tannins did not affect the total counts of protozoa and methanogens in the rumen.

The APL and APW diets increased the populations of *Ruminococcus albus*, *Ruminococcus flavefaciens* and *Fibrobacter succinogenes* in the rumen of goats. This finding could be due to the higher ruminal pH, which encouraged the proliferation of the microbes. This observation could be responsible for the improved nutrient digestibility in the AP goats. Contrarily, Singh et al. [24] observed that dietary Pakar leaves had no effect on the population of total fungi and bacteria and *Fibrobacter succinogenes* but decreased the population of *Ruminococcus flavefaciens*. Thus, the current results suggest that the *Ruminococcus albus*, *Ruminococcus flavefaciens* and *Fibrobacter succinogenes* were able to tolerate the level of polyphenols present in the APL and APW [9].

## Conclusion

The results of this study indicated that dietary APL and APW increased rumen pH and the concentration of propionate and reduced acetate concentration in goats. Dietary supplementation of AP enhanced the population of *Ruminococcus albus*, *Ruminococcus flavefaciens* and *Fibrobacter succinogenes* in the rumen and improved nutrient digestibility in goats. Further study to examine the impact of APL and APW on other groups of rumen microflora particularly those involved in lipid metabolism, and synthesis of other biohydrogenation intermediates is suggested.

## Abbreviations

AP: *Andrographis paniculata*
APL: *Andrographis paniculata* leaves
APW: *Andrographis paniculata* whole plant
FA: Fatty acid
PUFA: Polyunsaturated fatty acid
VFA: Volatile fatty acid
